# Placental Findings in Preterm and Term Preeclampsia: An Integrative Review of the Literature

**DOI:** 10.1055/s-0041-1730292

**Published:** 2021-08-30

**Authors:** Luciana Pietro, José Paulo de Siqueira Guida, Guilherme de Moraes Nobrega, Arthur Antolini-Tavares, Maria Laura Costa

**Affiliations:** 1 Institute of Health Sciences, Universidade Paulista, Campinas, SP, Brazil; 2 Department of Obstetrics and Gynecology, Universidade Estadual de Campinas, Campinas, SP, Brazil; 3 Department of Pathology, Universidade Estadual de Campinas, Campinas, SP, Brazil

**Keywords:** placenta, maternal mortality and morbidity, preeclampsia early onset, preeclampsia late onset, anatomopathological characteristics, placenta, mortalidade e morbidade materna, início precoce da pré-eclâmpsia, início tardio da pré-eclâmpsia, características anatomopatológicas

## Abstract

**Introduction** Preeclampsia (PE) is a pregnancy complication associated with increased maternal and perinatal morbidity and mortality. The disease presents with recent onset hypertension (after 20 weeks of gestation) and proteinuria, and can progress to multiple organ dysfunction, with worse outcomes among early onset preeclampsia (EOP) cases (< 34 weeks). The placenta is considered the root cause of PE; it represents the interface between the mother and the fetus, and acts as a macromembrane between the two circulations, due to its villous and vascular structures. Therefore, in pathological conditions, macroscopic and microscopic evaluation can provide clinically useful information that can confirm diagnosis and enlighten about outcomes and future therapeutic benefit.

**Objective** To perform an integrative review of the literature on pathological placental findings associated to preeclampsia (comparing EOP and late onset preeclampsia [LOP]) and its impacts on clinical manifestations.

**Results:** Cases of EOP presented worse maternal and perinatal outcomes, and pathophysiological and anatomopathological findings were different between EOP and LOP placentas, with less placental perfusion, greater placental pathological changes with less villous volume (villous hypoplasia), greater amount of trophoblastic debris, syncytial nodules, microcalcification, villous infarcts, decidual arteriolopathy in EOP placentas when compared with LOP placentas. Clinically, the use of low doses of aspirin has been shown to be effective in preventing PE, as well as magnesium sulfate in preventing seizures in cases of severe features.

**Conclusion** The anatomopathological characteristics between EOP and LOP are significantly different, with large morphological changes in cases of EOP, such as hypoxia, villous infarctions, and hypoplasia, among others, most likely as an attempt to ascertain adequate blood flow to the fetus. Therefore, a better understanding of the basic macroscopic examination and histological patterns of the injury is important to help justify outcomes and to determine cases more prone to recurrence and long-term consequences.

## Resumo

**Introdução** A pré-eclâmpsia (PE) é uma complicação da gravidez associada ao aumento da morbidade e mortalidade materna e perinatal. A doença se apresenta com hipertensão de início recente (após 20 semanas de gestação) e proteinúria, que pode progredir para disfunção de múltiplos órgãos, com resultados piores entre os casos de início precoce (<34 semanas). A placenta é considerada a principal causa da PE, representando a interface entre a mãe e o feto, e atuando como uma macromembrana entre as duas circulações, devido às suas estruturas vilosas e vasculares, de modo que, em condições patológicas, avaliações macroscópicas e microscópicas podem fornecer informações clinicamente úteis, que podem fornecer diagnóstico, prognóstico e benefício terapêutico.

**Objetivo** Realizar uma revisão integrativa da literatura para compreender e descrever os achados placentários patológicos associados à pré-eclâmpsia e seus impactos nas manifestações clínicas.

**Resultados** Os casos de início precoce apresentaram piores desfechos maternos e perinatais, e os achados fisiopatológicos e anatomopatológicos foram diferentes entre as placentas de início precoce e início tardio, com menor perfusão placentária, maiores alterações patológicas placentárias com menor volume viloso (hipoplasia vilosa), maior quantidade de debris trofoblásticos, nódulos sinciciais, microcalcificação, infartos vilosos, arteriolopatia decidual em placentas de início precoce quando comparadas com placentas de início tardio. Clinicamente, o uso de baixas doses de aspirina tem se mostrado significativo na prevenção da PE, assim como o sulfato de magnésio na prevenção de convulsões na doença com manifestações de gravidade.

**Conclusão** As características anatomopatológicas entre a pré-eclâmpsia precoce e tardia são significativamente diferentes, com grandes alterações morfológicas nos casos de início precoce, como hipóxia, infartos vilosos e hipoplasia, entre outros, na tentativa de estabilizar o fluxo sanguíneo para o feto. Portanto, um entendimento comum do exame macroscópico básico e dos padrões histológicos da lesão é importante para maximizar o benefício diagnóstico, prognóstico e terapêutico do exame da placenta e, consequentemente, reduzir os riscos para a mãe e o feto.

## Introduction

Hypertensive disorders in pregnancy, and in particular preeclampsia (PE) and eclampsia, are one of the three main causes of maternal mortality and morbidity globally, and an important cause of fetal and perinatal complications, such as increased risk of stillbirth, neonatal death, intrauterine growth restriction, and preterm childbirth. 1 Although there is conflicting information on the incidence of PE in different settings, it is estimated to affect ∼ 5 to 10% of pregnancies. According to a World Health Organization (WHO) review, PE is responsible for ∼ 16% of deaths in high-income countries, 9% of maternal deaths in Africa and Asia, and 25% in Latin America and the Caribbean. 2 In Brazil, the incidence of PE is probably underreported, varying from 1.5 to 7.5%, 3 with a rate of 0.6% for eclampsia. 4 According to Giordano et al., 5 in a multicenter study conducted in Brazil through the human development index (HDI), there was a prevalence of 5.2 cases of eclampsia per 1,000 live births, ranging from 2.2:1,000 in more developed areas to 8.3:1,000 in less developed areas, with a severe maternal outcome (death or near miss) resulting from eclampsia 5 times greater than that resulting from other serious complications related to hypertensive disorders of pregnancy. 6 7

**Fig. 1:**
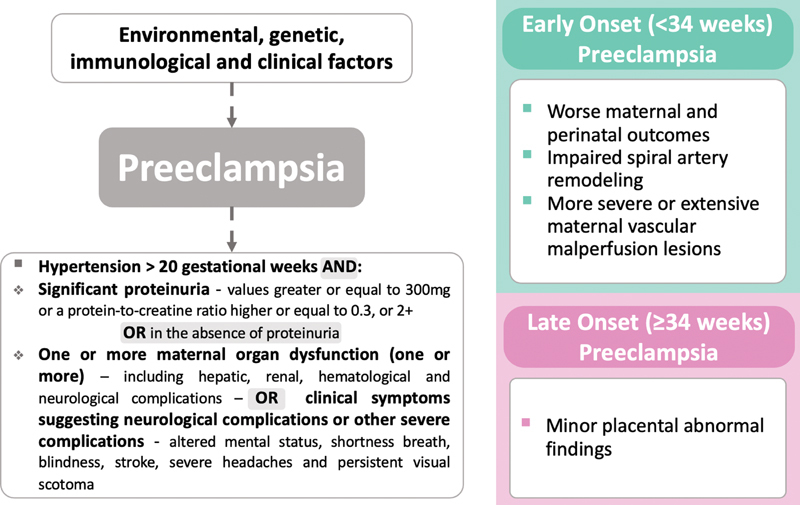
Definition of preeclampsia and classification according to the moment of diagnosis

Preeclampsia is a complex multifactorial syndrome, and its etiology has not yet been fully established, with influences of genetic-environmental interactions. 8 9 The definition of PE has been recently updated and, according to the International Society for the Study of Arterial Hypertension in Pregnancy (ISSHP), 10 to the American College of Obstetrics and Gynecology (ACOG) 9 , and endorsed by Brazilian recommendations, 6 PE occurs when a pregnant woman has hypertension (systolic blood pressure ≥ 140 mmHg and/or diastolic blood pressure ≥ 90 mmHg) accompanied by significant proteinuria (values ≥ 300mg in a 24-hour sample; or a protein-to-creatine ratio ≥0.3, or 1+ in urinalysis of patients proven to be without urinary tract infection), or, in the absence of proteinuria, ≥ 1of the following evidences of organic dysfunction: hepatic (with transaminases in concentration at least twice as high as the reference), impaired renal function (creatinine ≥1.2mg/dL), thrombocytopenia (with platelets < 100,000/mL or lactate dehydrogenase > 600 U/L; or clinical symptoms suggesting neurological complications (including altered mental status, blindness, stroke, severe headaches, and persistent visual scotoma), according to Fig. 1 . 6 8 9 11 12 13 14

The severity of adverse outcomes is strongly associated with gestational age at diagnosis, with late onset (≥ 34 weeks) associated with better maternal and perinatal outcomes, while the early onset (<34 weeks) of preeclampsia during pregnancy often leads to unfavorable outcomes, with increased placental abnormal findings, recurrence, and long-term consequences. 4

Disorders in the placenta are recognized as events that can compromise maternal and fetal health. 15 16 Different hypotheses of pathophysiological processes have been raised to justify the severity of the disease and its different clinical presentation during pregnancy. 17 The placenta is the fundamental organ in this process, since it is the only organ that presents a maternal and fetal interface, promoting almost all physiological interactions between the mother and the fetus, as the only source of oxygen, nutrition, and protection for the fetus. 18

The development of early onset PE is associated with poor placentation and dysfunction of the remodeling of the spiral arteries; those conditions are, however, uncommon in pregnancies affected by late PE, which usually do not present placental dysfunction. In addition, evidence of poor placentation is not an exclusive condition of PE and can also be recognized in pregnancies with fetal growth restriction, for example. 19

Preeclampsia is always a condition that needs constant awareness and follow-up, since clinical and laboratorial surveillance can detect early signs of severity that may increase maternal and perinatal risks. 10

In order to understand and describe the pathological placental findings associated to PE and its impacts on the clinical manifestations of the disease, we performed an integrative review of the literature on this issue.

## Primary Role of the Placenta

The placenta is a complex organ, composed of different types of cells, involving many functions, such as adhesion, invasion, vascular remodeling, cell fusion, hormonal production, and nutrient and waste transport. During pregnancy, the placenta plays a key role in providing a safe and protective environment in which the fetus can thrive, stimulating its own development. An intense change of substances involving the basal decidua and the endometrial glands provides the conceptus with an abundant source of energy, with rich secretions in carbohydrates and lipids. A failure in this process could compromise the development of cytotrophoblastic cells. The placenta also has an important function on the excretion of toxic fetal substances, and there is an intense interchange of antibodies and cells between the maternal and fetal circulation. 8 20 21 22

Under normal conditions, the formation of the placenta begins with the implantation of the blastocyst in the endometrium. 23 24 At this early stage, the outer cell mass of the human blastocyst is linked with trophoblastic cells that, upon contact with the endometrium, proliferate and differentiate into individualized cytotrophoblasts and a syncytial mass called syncytiotrophoblast. It is through the invasive properties of the syncytiotrophoblast that placental formation occurs until the 20 ^th^ week of pregnancy. Like the syncytiotrophoblast, the extra villous trophoblast cells (EVT) are also formed from cytotrophoblast proliferation, becoming invasive cells, characterized by the expression of human leukocyte antigen G (HLA-G). In this process, EVTs migrate through the deciduous stroma towards the spiral arteries, which are terminal branches of the uterine vessels within the endometrium, for the development of the placenta. 25 26 27

In an initial phase, the formation of syncytiotrophoblasts from the proliferation of cytotrophoblasts and fusion of newly formed cells at the interface with the maternal organism prevails, leading to the uneven growth of this cell layer. This is followed by a marked proliferation of cytotrophoblast cells that do not associate with each other and that, along with the underlying extra-embryonic mesoderm, enters the syncytiotrophoblast plates, forming three-dimensional filiform structures, called chorionic villi. These structures are much more developed on the maternal face. 23 24

During the differentiation of chorionic villi, the proliferation of cytotrophoblast cells continues in the apical regions of the villi. In these places, cytotrophoblastic cells make their way through the syncytiotrophoblast, reaching the endometrial stroma and forming the anchoring villi, followed by the formation of cell columns that detach from the basement membrane and invade the endometrial stroma is observed. Together, these cells are called extravillous cytotrophoblasts, responsible for: (1) locating the distal branches of the uterine arteries, called spiral arteries, and interacting with their smooth endothelial and muscle cells; and (2) to produce signaling molecules for maternal-placental communication in its different aspects (immunological, inflammatory, endocrine, and metabolic) and, in this way, mediate adaptive maternal responses to pregnancy. 24

**Fig. 2:**
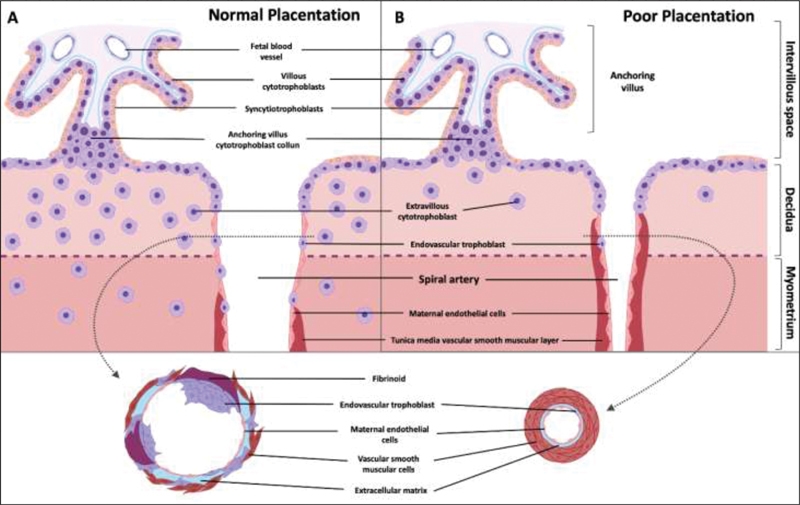
Diagrammatic representation of the effects of spiral artery remodeling on the inflow of maternal blood into the intervillous space in normal and pathological pregnancies (poor placentation)

In the absence of pregnancy, spiral arteries contain extensive smooth muscles that respond quickly to endocrine and vasoactive stimuli. During pregnancy, the EVTs destroy smooth muscle and elastin, replacing it with inert fibrinoid material. 28 Thus, the endovascular trophoblast reach the lumen of the spiral arteries, forming aggregates of cells that effectively obstruct the arteries during the first weeks of pregnancy. 29 Eventually, interstitial EVTs move through the stroma to reach the inner third of the myometrium ( Fig. 2 ).

These events in the maternal capillaries occur to contribute to the formation of a direct communication between the syncytiotrophoblast and the maternal blood. The contact of maternal blood with the surface of the trophoblast also seems to induce the formation of gaps in the syncytiotrophoblast. The growth and communication between chorionic villi form a tortuous network of channels through which maternal blood percolates. 8

**Fig. 3:**
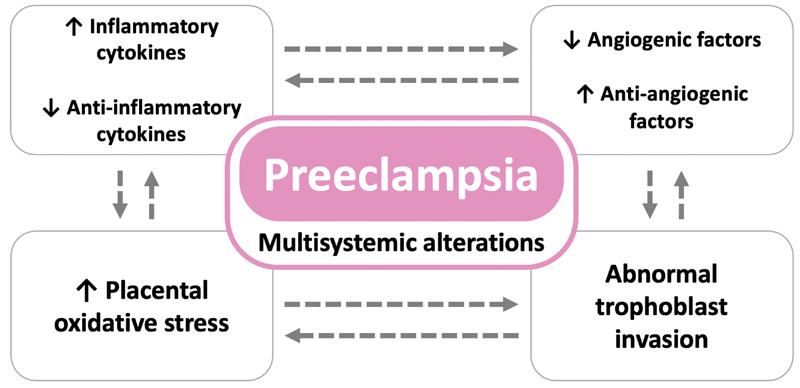
Underlying conditions of placental insufficiency leading to preeclampsia and fetal growth restriction. Numerous factors are involved in the development of the placenta under physiological conditions: pro- and antiangiogenic factors, pro- and anti-inflammatory cytokines, and pro- and antiapoptotic factors. Under pathological conditions, the expression of many of these factors is altered **Source:** Courtesy of A. Antolini-Tavares, São Paulo, Brazil.

However, there are some conditions in which this remodeling is compromised, as in PE, in which a defect in the implantation in the maternal uterine wall leads to decreased placental perfusion and intermittent blood flow, generating repeated episodes of ischemia-reperfusion, which triggers a favorable environment for the development of oxidative stress. As a result, free radicals lead to an inflammatory process in the placenta, to apoptosis, and to the release of cellular debris into the maternal circulation, along with many antiangiogenic factors, cytokines, and oxidants ( Fig. 3 ). 22 30 31

## Placental Development in Preeclampsia

Failures in the interaction between the decidua and endometrial tissue can lead to most reported placental complications. In cases in which there is severe underdevelopment of the trophoblastic cell layer, its association with spontaneous abortions is observed, while in less severe cases, in which there is compatibility with the continuation of pregnancy, there is a predisposition to conditions such as PE. 25 26

In the case of gestational complications such as PE, some authors describe that there is a failure in the destruction of the arterial walls in the EVT invasion process, triggering a restricted remodeling (shallow placement) with abnormal uteroplacental perfusion and placental dysfunction with excessive release of placental factors in the maternal circulation. 32 33 Therefore, an exaggerated maternal inflammatory response occurs, causing endothelial dysfunction, maternal hypertension, proteinuria, and other characteristics.

It is known that, in normal pregnancies, the presence of systemic inflammation is a characteristic; however, coexisting with another comorbidities. 34 In this process, these other comorbidities themselves can contribute to inflammation; so that it becomes difficult to distinguish cause and effect. 35

In PE, greater systemic inflammation occurs. 34 35 Once an inflammatory process is established, it results in atherosis, lesions characterized by fibrinoid necrosis and accumulation of macrophages loaded with lipids (foam cells), which are not restricted to the placental bed. Those lesions can appear virtually in any maternal blood vessel, and its clinical manifestation will be related to the most affected organ. 36 Unlike defective remodeling, atherosis can severely restrict the caliber of the uteroplacental vessels, causing secondary thrombosis lesions, limiting blood flow to the placenta, and causing infarctions with a risk of fetal death. 37 Also, this process will contribute to the continuous release of free radicals formed after a sequence of ischemia-reperfusion processes.

Thus, it is known that placental lesions in PE reflect mainly poor perfusion, such as infarctions in the placental villi, regardless of the stage of development, absence of villi, fibrin deposition, reperfusion lesions, and inflammation. According to Sebire, 38 these lesions are not specific conditions of the PE syndrome, but they are between four and seven times more frequent in these conditions when compared with normotensive pregnancies.

At the microscopic level, there is focal necrosis of the syncytiotrophoblast, with loss and distortion of microvilli; 39 40 hyperplasia around cytotrophoblastic cells may be present with degeneration or apoptosis of some cells. 41

## Main Placental Morphological findings in Preeclampsia

There has been much heterogeneity in the style and quality of placental pathology reports. Recently, attempts have been made to encourage the standardization of the terminology, through the Amsterdam Placental Workshop Group Consensus Statement, with the aim of improving the comparability and quality of reports of placental pathology, through defined diagnostic criteria. 18 42 43

One of the standardizations is the inclusion of the weight of the placenta and of observations about its size, whether it is small or large for the declared gestational age. Clinically significant gross findings can also be integrated into the diagnosis, especially if there are histological correlates and if the gross lesions support a specific diagnostic category. 18 44

In this regard, it is important to check whether the placenta is complete or whether it is torn or fragmented, to the point that it cannot be reconstructed, or if cotyledons are missing. In this case, the placenta is considered incomplete, and the possibility of retained placental tissue should be raised and managed properly. The maternal surface should also be assessed for the presence and size of any adherent blood clot, which may represent evidence of placental detachment. 18

Another important analysis is related to placental lesions associated with altered maternal perfusion, caused mainly by defects in trophoblast invasion and remodeling of maternal spiral arterioles. 45 46 Before the Amsterdam Consensus, the placental lesions in PE could have many names, such as placental ischemia, maternal vascular underperfusion, placental insufficiency, and Tenney-Parker changes, which do not better reflect the lesions pathogenesis. Now, maternal vascular malperfusion (MVM) can conceptually include this, and it can be separated into two categories of histological findings: global/partial (partial interruption of perfusion across the placenta, as infarcts), and segmental/complete (complete interruption of blood flow to a portion/segment of the placenta, as distal villous hypoplasia and/or patchy accelerated villous maturation). Usually, both patterns are present.

Maternal vascular malperfusion placentas are generally small for gestational age (< 10 ^th^ centile), and have an increased or “hypoplastic” fetal/placental weight ratio. The umbilical cord can be thin, and there is often a villous infarction, represented by firm lesions, yellowish-white to yellowish-yellow, within the placental parenchyma. Although infarctions are often best seen after formalin fixation, they can usually be felt as firm areas, even though they are obviously not visible in fresh placentas. 18

**Fig. 4:**
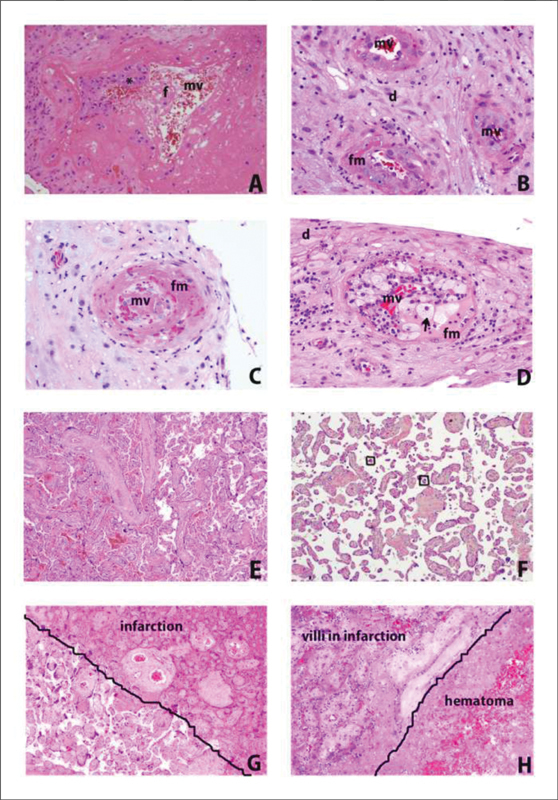
Representative morphological findings in placentas at term and early onset preeclampsia (EOP) cases – Hematoxylin and Eosin (H/E) analysis. In **A,** we can observe the invasion of the trophoblast (*) to transform maternal vessels (mv) from high pressure to low pressure, with physiological formation of fibrin (f), at the beginning of pregnancy (HE, 20x objective). In **B** , it is possible to observe the vessels of the decidua (d) with an evident and thick (hypertrophic) smooth muscle layer, with small intramural deposits of fibrinoid material (fm) (HE, 20x objective). In **C** , we observe the decidual vessel with extensive destruction of the muscle layer (necrosis) and replacement with fibrinoid material (fm) (HE, 20x objective). In **D** , it is possible to observe the vessel with necrosis of the muscle layer and inflammatory recruitment of macrophages (foamy cytoplasm) (arrows) in the middle of fibrinoid material (fm), characterizing atherosis (HE, 20x objective). In **E** , we observed a normal placenta at term, without distal villous hypoplasia (HE, 2.5x objective). In **F** , a case of EOP, we have a panoramic view of thin and elongated chorionic villi, with small and miniaturized terminal villi (within the square), with fewer vessels inside (usually one or two), in relation to mature villi (about three vessels in each terminal villus), characterizing distal villous hypoplasia (HE, 4x objective). In **G** , a case of EOP, it is possible to observe infarction centrally located in the placental parenchyma, due to hypertension in the vascular bed of the intervillous space, associated with the interaction between trophoblast and deficient uterine vessels: it is observed that, on the right, the villi are agglutinated (decreasing the distance between them due to an abrupt decrease in blood in the intervillous space) and a more eosinophilic color (pink) with less distinction of cell morphology (necrosis), and that, in the lower left corner, the villi have a more mature pattern, as if there were a “penumbra” of hypoxia that was not able to cause ischemic cell death (HE, 4x objective). And in **H** , a case of EOP, we observed another feature of hypertensive placental infarction, this time associated with a hematoma inside (hematoma infarction) (HE, 10x objective).

Microscopically, it is possible to observe mural hypertrophy, which refers to a concentric thickening of the vessel wall, to the point that the vessel lumen occupies ≤ 30% of the total diameter of the arteriole, a process called decidual arteriopathy. This lesion can progress to early degenerative changes in the wall, associated with a chronic perivascular inflammatory infiltrate, called chronic perivasculitis, and fibrinoid necrosis of the arteriolar wall, with or without acute atherosis ( Fig. 4 ). 37 46

These main findings showed in Fig. 4 are also reported in Chart 1 , with a selection of studies that presented the pathophysiological and anatomopathological characteristics in early and late onset PE placentas, through a comprehensive search of the publications in the Google Scholar, PubMed, and MEDLINE databases.

**Chart 1:** Summary of the main changes in pathophysiological and anatomopathological findings when comparing placentas of early and late onset preeclampsia

Author/year	N° participants	Evaluated cases/groups	Main Findings
Shchegolev et al. (2016) 47	138	28 control group 26 EOP 84 LOP	Preponderance of branching angiogenesis in the preeclamptic chorionic villi and an increase in the number of syncytial nodules in EOP and microcysts in the septae in LOP. Morphometric analysis of immunohistochemical placental specimens established increased signs of placental hypoxia in EOP.
Orabona et al. (2016) 48	177	105 EOP 72 LOP	Increased incidence of distal villous hypoplasia, decidual arteriolopathy, syncytiotrophoblast ‘knots’, microcalcification, villous infarcts, perivillous fibrosis, intervillous thrombi and nonspecific inflammatory lesions in the EOP
Khodzhaeva et al. (2016) 49	150	50 control group 50 EOP 50 LOP	Greater remodeling of spiral arteries in EOP; with increased incidence of sclerosed blood vessel and lower incidence of intact vessels and smooth muscle cells in EOP compared to LOP. The degree of compensation of chronic hypoxia tissue in the area of the placental site was typical for LOP and was absent of an EOP.
Zhang et al. (2015) 50	178	54 EOP 124 LOP	Decidual vascular disease, placental infarction, abruptio placentae and placental villi dysplasia were seen in both groups. The incidence of decidual vascular disease and placental villi dysplasia was higher in EOP, and the incidence of placental infarction was lower when compared with LOP.
Kovo et al. (2012) 51	130	37 EOP 93 LOP	EOP had higher rates of FGR and lesions of maternal vascular supply. Within the LOP group, cases with FGR had higher rates of maternal vascular supply lesions than those without FGR, but similar rates of fetal vascular supply lesions
Ogge et al. (2011) 52	19.041		Prevalence of lesions consistent with maternal underperfusion in EOP and LOP, but in EOP groups had a significantly higher frequency of placental lesions when compared with LOP.
Van der Merwe et al. (2010) 53	100	50 control group 25 EOP 25 LOP	EOP presented increased incidence of pathological infarction and chorionic plate thrombosis, however the LOP presented more incidence of decidual arteriopathy.
Egbor et al. (2006) 54	69	9 EOP 11 LOP	EOP presented hipoplasic villi volume and impairment of total surface area of the terminal villi, while in LOP there was impact on peripheral villi or vasculature features

Abbreviations: EOP, early onset preeclampsia; FGR, fetal growth restriction; LOP, late onset preeclampsia.

In global/partial MVM, accelerated villous maturation is observed microscopically, referring to regions of the placenta with underdevelopment and villous scarcity, alternating with regions with villous agglomeration, with increased syncytial nodes, perivillous fibrin deposition, and villous agglutination. 9 Consequently, there is the presence of small or short hypermature villi for gestational age, reflecting the narrowing of the uterine arteries caused by defective remodeling and decidual arteriopathy, leading to uneven maternal perfusion. 43

In the case of segmental/complete MVM, it the presence of villous infarctions is microscopically observed, with well-defined margins and usually located in the basal plate, representing a region of the placenta that has suffered necrosis due to the complete loss of maternal perfusion. Depending on the age of the infarction, the trophoblast loses nuclear basophilia, with luminal destruction of the fetal vessels, stromal fibrosis, and complete involution of the trophoblast and fetal vessels, triggering collapse of the intervillous space, and villi surrounded by fibrin. 18

## Relevance of Anatomopathological and Clinical Analysis

The placenta, in representing the interface between the mother and the fetus, acts as a macromembrane between the two circulations: placental-fetal and placental-maternal. Due to their villous and vascular structures, under pathological conditions, macroscopic and microscopic examinations can provide clinically useful information, which can maximize the diagnosis, prognosis, and therapeutic benefits. 18 55

Among the main benefits of the placental exam, we can consider the identification of etiologies and pathological processes that may contribute or explain an adverse result in pregnancy, as well as better management of subsequent pregnancies by identifying conditions known to be at risk of recurrence or that may be treatable or preventable. 55 Since the placenta is the common link between obstetricians, neonatologists and pathologists, it is important to try together to understand the maternal, fetal, and neonatal physiological aspects, as well as the pathological processes that determine the outcomes of pregnancies. A common understanding among all parties regarding the indications, the basic examination and the histological patterns of the lesion are important to maximize the diagnosis, the prognosis, and therapeutic benefits of the placental examination. 18

In the histology field, strategies to prevent PE have been studied extensively in the past 30 years. However, until now, no intervention has proven to be fully effective to prevent PE.

Among these strategies, the most studied is the use of aspirin. Numerous studies have investigated its relationship in the prevention of PE, since there are hypotheses that relate the development of PE to an imbalance in the metabolism of prostacyclin and thromboxane A _2_ involved in the pathogenesis of PE. Therefore, the administration of low doses of aspirin starting at the 16 ^th^ week of gestation would be able to inhibit the troboxane A _2_ . However, randomized studies showed only a modest reduction in the prevention of the development of PE, different from what was observed when the administration started before the 16 ^th^ week of pregnancy. In these cases, there was a significant reduction in cases of severe PE and intrauterine growth restriction. 6 9 56

In view of these data, the ACOG 9 advises that women with any of the high risk factors for PE (previous pregnancy with PE, multifetal pregnancy, kidney disease, autoimmune disease, type 1 or type 2 diabetes mellitus, and chronic hypertension), and those with > 1 of the moderate risk factors (1 ^st^ pregnancy, maternal age ≥ 35 years old, PE family history, sociodemographic characteristics, and personal history factors) should receive low doses aspirin for PE prophylaxis starting between 12 and 28 weeks of gestation (ideally before 16 weeks of gestation), and continuing until delivery. The Brazilian guideline on PE also endorses this recommendation, and due to the availability in our scenario, the proposed dose is 100mg/day, preferably at night. 6 In settings with low calcium consumption (such as Brazil), another proven strategy in the prevention of PE is the use of 1.5g calcium/day during gestation. 9

Another important strategy in preventing severe features is the administration of magnesium sulfate. Magnesium sulfate has been used in obstetrics since 1925 to prevent and control seizures in specific hypertensive diseases of pregnancy, with the advantage of decreasing peripheral vascular resistance without altering uterine blood flow, due to its anticonvulsant properties, through blocking of the N-methyl-D-aspartate (NMDA) receptor and calcium channels, with its use being recommended by the ACOG and the WHO, as well as by national guidelines, 6 as it is safer and more effective than phenytoin, diazepam and lytic cocktail (chlorpromazine, promethazine, and pethijantar) in preventing recurrent seizures in eclampsia, in addition to being low cost and easy to administer. 6 57 58

Research has attested to the effectiveness of magnesium sulfate in preventing seizures in women with severe PE and eclampsia, proving that its administration can halve the risk of eclampsia, of placental detachment and, consequently, the risk of maternal death. Treatment of PR is based on accurate diagnosis, with fetal assessment, blood pressure control, and decision on the timing of delivery. Delivery is indicated at any gestational age if severe features and at term if there are no signs of severity. 6 Removal of the placenta is still the most effective intervention. However, the disease does not end with childbirth. Postpartum can be critical for blood pressure control and clinical symptoms and complications, especially in the first few days. Women with a history of PE, and mostly those with early onset, should be closely follow-ed up and receive adequate counseling on the future risk of recurrence and long-term complications.

## Conclusion

The anatomopathological characteristics between early and late PE are significantly different with large morphological changes in cases of early onset PE, such as hypoxia, villous infarctions, and hypoplasia, among others, in an attempt to stabilize the blood flow to the fetus. Therefore, a better understanding of the basic macroscopic examination and histological patterns of the injury is important to help justify outcomes and to determine cases more prone to recurrence and long-term consequences.
